# A Highly Selective and Non-Reaction Based Chemosensor for the Detection of Hg^2+^ Ions Using a Luminescent Iridium(III) Complex

**DOI:** 10.1371/journal.pone.0060114

**Published:** 2013-03-22

**Authors:** Daniel Shiu-Hin Chan, Wai-Chung Fu, Modi Wang, Li-Juan Liu, Chung-Hang Leung, Dik-Lung Ma

**Affiliations:** 1 Department of Chemistry, Hong Kong Baptist University, Kowloon Tong, Hong Kong, China; 2 State Key Laboratory of Quality Research in Chinese Medicine, Institute of Chinese Medical Sciences, University of Macau, Macao, China; Queen's University Belfast, United Kingdom

## Abstract

We report herein a novel luminescent iridium(III) complex with two hydrophobic carbon chains as a non-reaction based chemosensor for the detection of Hg^2+^ ions in aqueous solution (<0.002% of organic solvent attributed to the probe solution). Upon the addition of Hg^2+^ ions, the emission intensity of the complex was significantly enhanced and this change could be monitored by the naked eye under UV irradiation. The iridium(III) complex shows high specificity for Hg^2+^ ions over eighteen other cations. The system is capable of detecting micromolar levels of Hg^2+^ ions, which is within the range of many chemical systems.

## Introduction

Mercury is a highly toxic heavy metal ion that is harmful to both humans and the environment. Metabolism by marine microorganisms converts mercury ions into methylmercury, a highly toxic and bio-accumulative form [1] that damages the human central nervous and endocrine systems and is associated with sensory, motor and cognitive disorders [2]. Evidence has also suggested that exposure to high levels of mercury ions can damage the lungs and kidneys [3]. Therefore, the development of new methods for the selective detection of mercury ions is of particular importance and remains an active area of research in the scientific community.

Traditional instrumental techniques for detection of Hg^2+^ ions include atomic absorption/emission spectrometry (AAS/AES) [4], [5], inductively-coupled plasma mass spectrometry or atomic emission spectroscopy (ICP-MS/ICP-AES) [6]–[8] and X-ray fluorescence (XRF) [9]–[11]. Despite their widespread usage in industry and the laboratory, these methods are time-consuming and require extensive pre-treatment procedures, and involve the use of complex and expensive instrumentation. Over the past decade, a number of alternative methods for the detection of metal ions have been reported, including luminescent chemosensors [12]–[19], electrochemical sensors [20], [21] and colorimetric probes [22]–[24]. However, most luminescent probes for Hg^2+^ ions only perform well in organic solvents [13], [17], [18], which is not favourable for real sample analysis. Therefore, it is desirable to develop water-soluble luminescent probes for Hg^2+^ ions that can function effectively in aqueous solution.

Luminescent transition metal complexes have attracted considerable attention in the fabrication of organic optoelectronics [25], [26], luminescent sensors [27]–[32] and cellular imaging [33]–[41] by virtue of their salient advantages: (i) the ^3^MLCT emission of many metal complexes lie in the visible spectral region, (ii) their long-lived phosphorescence emission can be resolved from a fluorescent background by time-resolved spectroscopic techniques, thus enhancing signal imaging stability, (iii) the significant stokes shifts of the complexes allow for easy separation of their excitation and emission wavelengths, thus preventing self-quenching, (iv) their facile colour-tuning ability makes them suitable for different photophysical applications [42]–[49], and (v) the preparation of metal complexes is highly modular.

**Figure 1:**
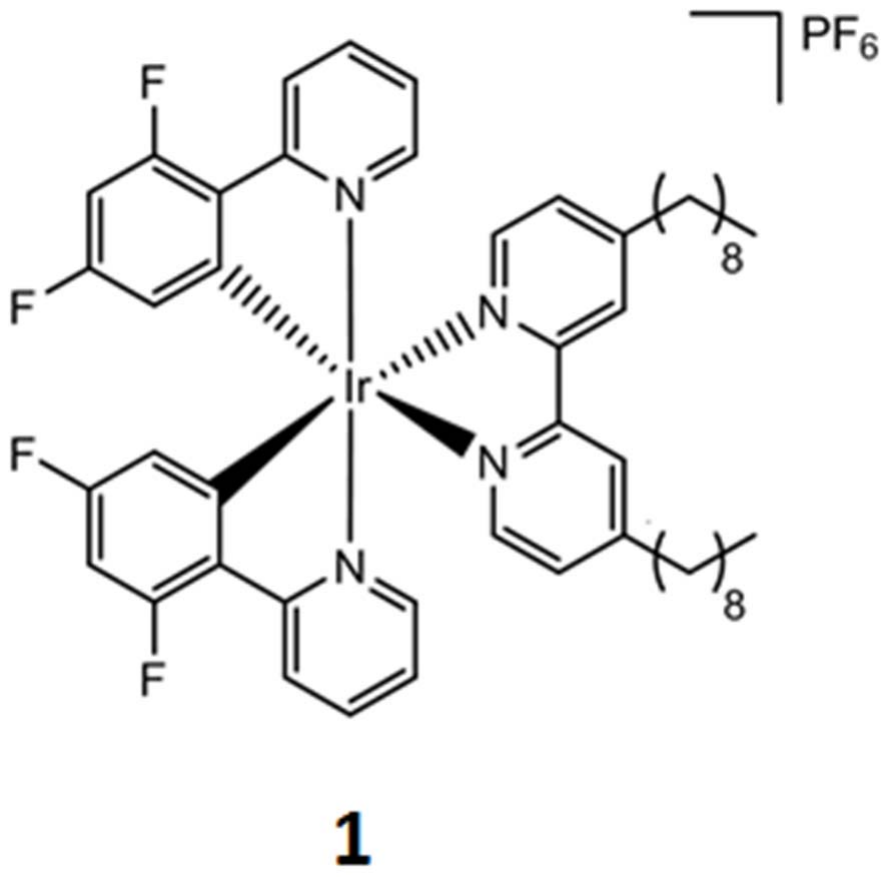
Chemical structure of the iridium(III) complex 1 bearing the 4,4’-dinonyl-2,2’-bipyridine ligand.

While luminescent iridium(III) complexes have been successfully applied in a variety of fields, there are few reports on luminescent iridium(III)-based chemosensors for the detection of Hg^2+^ ions. Li, Huang and co-workers reported an iridium(III) complex as a chemodosimeter of Hg^2+^ ions based on the interaction between Hg^2+^ and the sulfur atom of the cyclometalated ligands [50]–[52]. Lu and co-workers fabricated a chemosensor for Hg^2+^ ions based on the dissociation of a dithiocarbamate ligand from the iridium(III) complex [53]. However, these reaction-based iridium(III) chemosensors are strictly dependent upon the quantitative interaction between the metal complex ligands and Hg^2+^ ions. In this work, we report the application of a novel cyclometalated iridium(III) complex [Ir(dfppy)_2_(dnbpy)]^+^ (**1**, where dfppy = 2,4-difluorophenylpyridine and dnbpy = 4,4′-dinonyl-2,2′-bipyridine) (Figure 1) as a non-reaction based switch-on chemosensor for Hg^2+^ ions in aqueous solution.

## Results and Discussion

The photophysical properties of complex **1** are provided in Table S1, Figure S1 and S2. Complex **1** displays a strong absorption band between 250 and 300 nm which can be attributed to spin-allowed π-π* ligand-centered (^1^LC) transitions of the dfppy ligand [54]. The absorption band at 303 nm is assigned to an iridium-based spin allowed metal-to-ligand charge transfer (^1^MLCT) transition, while the low-energy absorption shoulder at approximately 450 nm is assigned to spin-forbidden triplet ^3^MLCT transitions according to the peak assignment of a similar iridium(III) complex [55]. The emission peak at λ = 490 nm is phosphorescent in nature as revealed by its relatively long emission lifetime (4.53 µs).

**Figure 2:**
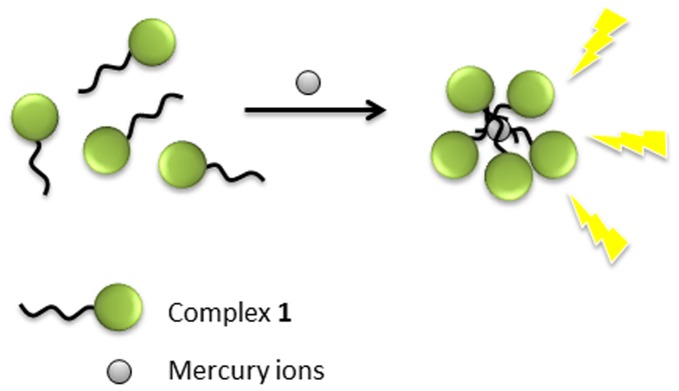
Schematic illustration of the non-reaction based assay for the detection of Hg^2+^ ions using luminescent iridium(III) complex 1. The adsorption of Hg^2+^ ions on the hydrophobic side chains induces aggregation of complex **1.** This partially shields the complexes from solvent interactions, hence resulting in an enhanced emission intensity at λ = 490 nm.

The luminescence response of **1** upon addition of different concentrations of Hg^2+^ ions was first investigated by emission titration experiments. Complex **1** was weakly emissive in aqueous buffered solution. However, the luminescence of **1** was significantly enhanced in the presence of the increasing concentrations of Hg^2+^ ions. We presumed that the unusual sensing behaviour of the complex towards Hg^2+^ may be due to the presence of its hydrophobic side chains, which are known to have a tendency to adsorb Hg^2+^ ions [56]. The possible sensing mechanism of this Hg^2+^ chemosensor is depicted in Figure 2. Mercury ions may interact with the hydrophobic carbon chains of multiple complexes, inducing aggregation of the iridium(III) complexes into a micelle-like motif. This results in a strong enhancement of the luminescence emission of **1** at λ = 490 nm, presumably due to the partial protection of the complex from non-radiative decay by solvent quenching, thus giving rise to an enhanced ^3^MLCT emission.

**Figure 3:**
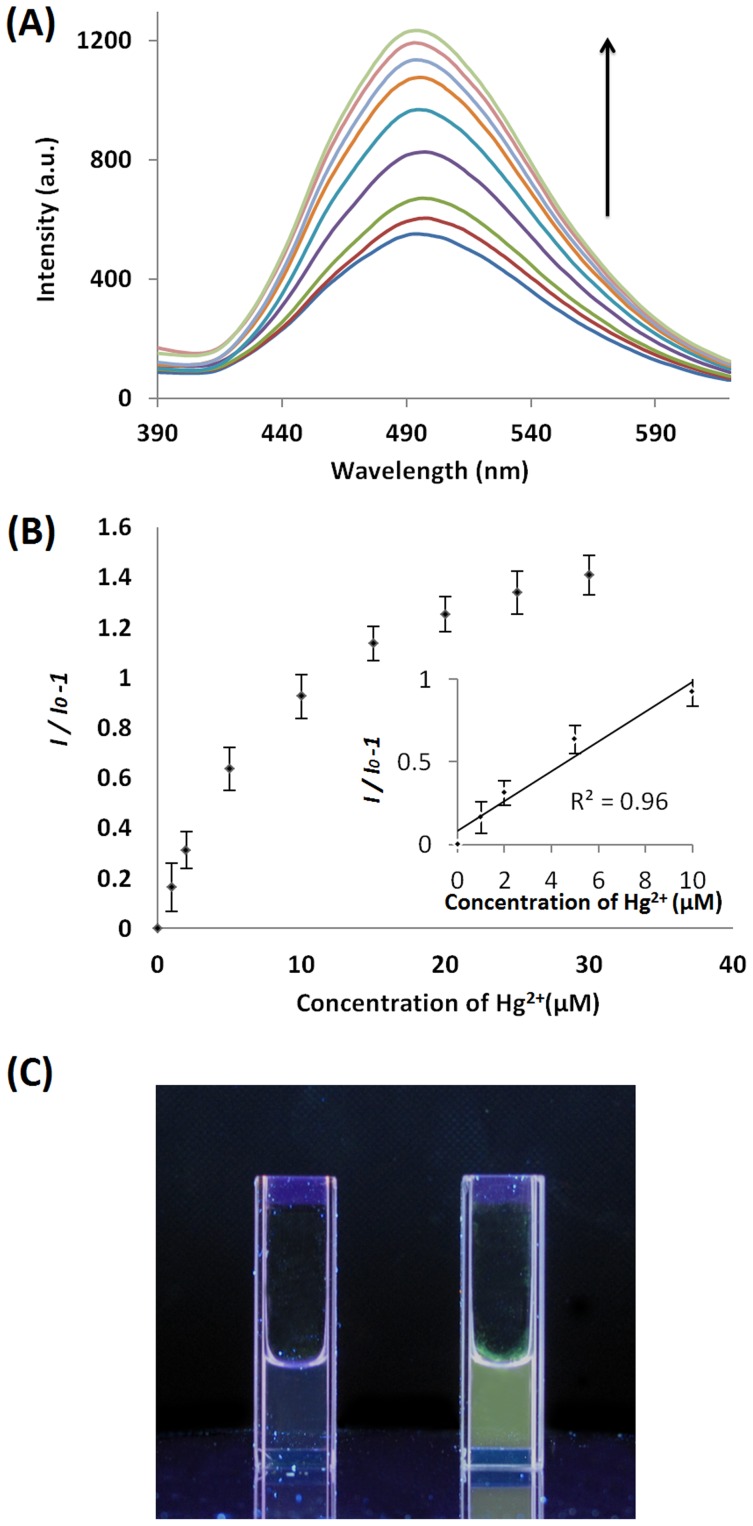
(Upper panel) Emission spectrum of complex 1 (0.5 µM) in the presence of increasing concentrations of Hg^2+^ ions (0, 1, 2, 5, 10, 15, 20, 25, 30 µM). (Middle Panel) Luminescence response of the system at λ = 490 nm *vs.* Hg^2+^ concentration. Inset: linear plot of the change in luminescence intensity at λ = 490 nm *vs.* Hg^2+^ concentration. Error bars represent the standard deviations of the results from three independent experiments. (Lower Panel) Photograph image of **1** (0.5** µ**M) in Tris buffer (25 mM, pH 7.0) in the absence (left) or presence (right) of 30** µ**M of Hg^2+^ ions under UV irradiation.

To optimize the performance of the system, we investigated the effect of the concentration of complex **1** on the luminescence response of the system to Hg^2+^ ions. The results showed that a concentration of 0.5 µM of complex **1** was optimal for this study, as the sensitivity and dynamic range of detection of the system were reduced at higher concentrations (Figure S3). Under the optimal conditions, we investigated the application of the proposed system for the detection of Hg^2+^ ions in aqueous buffered solution (25 mM Tris, pH 7.0). As shown in Figure 3, a strong increase in the emission intensity of **1** was observed upon addition of Hg^2+^ ions, with a maximum luminescence enhancement (*I*/*I*_0_ –1) of *ca.* 1.4 at saturating concentrations of [Hg^2+^]. A linear relationship was observed between the luminescence intensity of **1** and the Hg^2+^ concentration (R^2^ = 0.96) in the range of 0–10 µM of Hg^2+^ (Figure 3). The detection limit at a signal to noise ratio of 3 was found to be 2.8 µM, which is sufficient for the detection of Hg^2+^ ions in many chemical systems. The luminescence enhancement of the system upon the addition of micromolar Hg^2+^ ions can be readily observed by the naked eye under UV-irradiation (Figure 3). These results indicate that with a portable spectrophotometer, complex **1** could possibly be used in field studies as a sensitive “naked-eye” indicator for Hg^2+^ ions in water samples.

Thiol-containing compounds can effectively sequester Hg^2+^ ions by the formation of the strong Hg(II)–S bond, and this fact has been utilized in the fabrication of assays for detection of both bio-thiols and Hg^2+^ ions [57], [58]. To validate our hypothesis that the enhanced luminescence of **1** is due to the direct interaction between the metal complex and Hg^2+^ ions, we investigated the effect of adding cysteine to a solution of **1** and Hg^2+^ ions (Figure S4). The results showed that the emission intensity of **1** was significantly decreased upon the addition of cysteine, which could be attributed to the extraction of Hg^2+^ ions by the strong Hg(II)–S interaction and the subsequent dissociation of the metal complex aggregate. The interaction between **1** and Hg^2+^ ions was further examined by ^1^H NMR titration experiments in CD_3_CN solution (Figure S5). The aromatic protons of complex **1** were not significantly perturbed upon the addition of Hg^2+^ ions, indicating the absence of ligand replacement or covalent binding between Hg^2+^ ions and metal complex **1**, which is unlike the previously reported iridium(III) Hg^2+^ chemodosimeters reported [51], [53].

**Figure 4:**
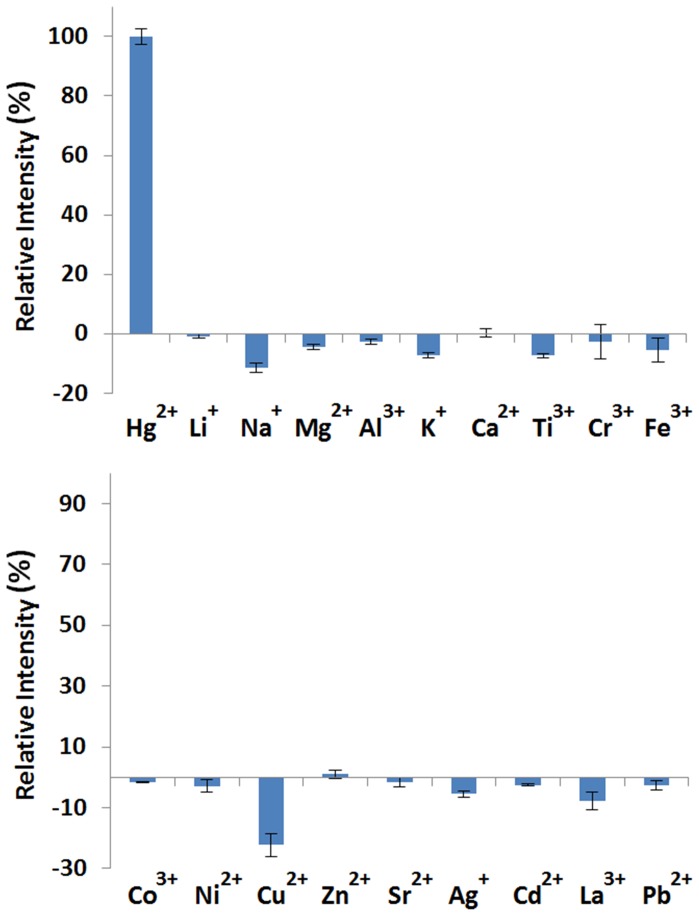
Luminescence response of complex 1 (0.5 µ µM) in the presence of Hg^2+^ (5 µM) or 10-fold excess of various metal ions (Li^+^, Na^+^, Mg^2+^, Al^3+^, K^+^, Ca^2+^, Ti^3+^, Cr^3+^, Fe^3+^, Co^3+^, Ni^3+^, Cu^2+^, Zn^2+^, Sr^2+^, Ag^+^, Cd^2+^, La^3+^, Pb^2+^) in Tris buffer (25 mM, pH 7.0). Error bars represent the standard deviations of the results from three independent experiments.

The specific response of the system to Hg^2+^ ions was evaluated by examining the luminescence signal of complex **1** in the presence of various metal ions under the optimal conditions. As shown in Figure 4, only the addition of Hg^2+^ could induce a prominent increase in the luminescence emission of **1**, whereas the addition of 10-fold of eighteen other cations (Li^+^, Na^+^, Mg^2+^, Al^3+^, K^+^, Ca^2+^, Ti^3+^, Cr^3+^, Fe^3+^, Co^3+^, Ni^3+^, Cu^2+^, Zn^2+^, Sr^2+^, Ag^+^, Cd^2+^, La^3+^, Pb^2+^) caused only very slight luminescence changes. The slight decrease in luminescence intensity upon the addition of 10-fold excess of certain metal ions may be presumably attributed to the disruption of pre-aggregation of **1** by those cations [54].

A competition study on the selectivity of **1** towards Hg^2+^ was also conducted to investigate the performance of the assay in the presence of interfering metal ions. The luminescence signal of the probe was slightly decreased upon addition of a mixture of five interfering metal ions (150 µM each of Pb^2+^, Fe^3+^, Co^2+^, La^3+^, Ti^3+^) (Figure S6). However, the subsequent addition Hg^2+^ (30 µM) strongly promotes the aggregation of **1** due to the specific binding of Hg^2+^ ions to the hydrophobic side chains of **1**, thereby enhancing its luminescence emission. This result demonstrates that the Hg^2+^ detection assay is able to function effectively even in the presence of multiple interfering metal ions at excess.

## Conclusions

In summary, we have synthesized and characterized a novel non-reaction based luminescent iridium(III) complex **1** for the rapid, selective and direct detection of Hg^2+^ in aqueous solution. This chemosensor displays a strong luminescence “switch-on” response to Hg^2+^ with a detection limit in low-micromolar range, which is comparable to existing iridium(III)-based Hg^2+^ chemosensors, and is highly selective for Hg^2+^ over eighteen other metal ions. Furthermore, the addition of cysteine to the system can revert the luminescence signal of **1** to the “off” state. We envisage this luminescent iridium(III) complex could be further developed as a reusable Hg^2+^ chemosensor for the sensitive detection of Hg^2+^ in aqueous solution.

## Materials and Methods

### Chemicals and materials

Reagents were purchased from Sigma Aldrich and used as received. Iridium chloride hydrate (IrCl3.xH2O) was purchased from Precious Metals Online.

### General experimental

Mass spectrometry was performed at the Mass Spectroscopy Unit at the Department of Chemistry, Hong Kong Baptist University, Hong Kong (China). Melting points were determined using a Gallenkamp melting apparatus and are uncorrected. Deuterated solvents for NMR purposes were obtained from Armar and used as received.

^1^H and ^13^C NMR were recorded on a Bruker Avance 400 spectrometer operating at 400 MHz (^1^H) and 100 MHz (^13^C). ^1^H and ^13^C chemical shifts were referenced internally to solvent shift (CD_3_CN:^ 1^H, δ1.94, ^13^C, δ118.7; d_6_-DMSO: ^1^H, δ2.50, ^13^C δ39.5). Chemical shifts (δ) are quoted in ppm, the downfield direction being defined as positive. Uncertainties in chemical shifts are typically ±0.01 ppm for ^1^H and ±0.05 for ^13^C. Coupling constants are typically±0.1 Hz for ^1^H-^1^H and ±0.5 Hz for ^1^H-^13^C couplings. The following abbreviations are used for convenience in reporting the multiplicity of NMR resonances: s, singlet; d, doublet; t, triplet; q, quartet; m, multiplet; br, broad. All NMR data was acquired and processed using standard Bruker software (Topspin).

Absorption spectra were recorded on a Cary 300 UV/Vis spectrometer. Emission spectra were recorded on a PTI QM4 spectrometer. Quantum yields and lifetime measurements were performed on a PTI TimeMaster C720 Spectrometer (Nitrogen laser: pulse output 337 nm) fitted with a 380 nm filter. Error limits were estimated: λ (±1 nm); τ (±10%); φ (±10%). All solvents used for the quantum yield and lifetime measurements were degassed using three cycles of Freeze-Vacuum-Thaw.

### Synthesis of [Ir(dfppy)_2_(dnbpy)]PF_6_

#### [Ir(dfppy)_2_(dnbpy)]PF_6_

A suspension of [Ir_2_(dfppy)_4_Cl_2_] [59] (120 mg, 0.1 mmol) and 4,4'-dinonyl-2,2'-bipyridine (89.8 mg, 0.22 mmol) in a mixture of dichloromethane:methanol (1:1, 20 mL) was refluxed overnight under a nitrogen atmosphere. The resulting solution was then allowed to cool to room temperature, and filtered to remove unreacted cyclometalated dimer. To the filtrate, an aqueous solution of ammonium hexafluorophosphate (excess) was added and the filtrate was reduced in volume by rotary evaporation until precipitation of the crude product occurred. The precipitate was then filtered and washed with several portions of water (2×50 mL) followed by diethyl ether (2×50 mL). The product was recrystallized by acetonitrile:diethyl ether vapor diffusion to yield the titled compound as a yellow-green solid.

Yield: 68%. ^1^H NMR (400 MHz, CD_3_CN) d 8.38 (s, 2H), 8.31(d, *J* = 8.0 Hz, 2H), 7.90 (t, *J* = 8.0 Hz, 2H), 7.82 (d, *J* = 4.0 Hz, 2H), 7.60 (d, *J* = 8.0 Hz, 2H), 7.35 (d, *J* = 8.0 Hz, 2H), 7.08 (t, *J* = 8.0 Hz, 2H), 6.68 (t, *J* = 8.0 Hz, 2H), 5.73 (d, *J* = 8.0 Hz, 2H), 2.81 (t, *J* = 8.0 Hz, 4H), 1.73–1.66 (m, 4H), 1.33–1.27 (m, 24H), 0.87 (t, *J* = 4.0 Hz, 6H); ^13^C NMR (400 MHz, CD_3_CN) d 166.3, 166.2, 165.3, 165.2, 164.2, 164.0, 163.8, 163.6, 161.6, 161.5, 158.3, 156.8, 156.3, 156.2, 151.6, 150.8, 140.8, 129.8, 129.4, 126.2, 125.2, 125.1, 125.0, 115.2, 115.0, 100.2, 100.0, 99.7, 36.3, 33.0, 31.2, 30.5, 30.4, 30.3, 30.2, 23.8, 14.8; MALDI-TOF-HRMS: Calcd. For C_50_H_56_F_4_IrN_4_ [M-PF_6_]^+^: 981.4068. Found: 981.4089

### Photophysical measurement

Emission spectra and lifetime measurements for complex **1** were performed on a PTI TimeMaster C720 Spectrometer (Nitrogen laser: pulse output 337 nm) fitted with a 380 nm filter. Error limits were estimated: λ (±1 nm); τ (±10%); φ (±10%). All solvents used for the lifetime measurements were degassed using three cycles of freeze-vac-thaw.

Luminescence quantum yields were determined using the method of Demas and Crosby [Ru(bpy)_3_][PF_6_]_2_ in degassed acetonitrile as a standard reference solution (Φ_r_ = 0.062) and calculated according to the following equation:



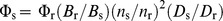
where the subscripts s and r refer to sample and reference standard solution respectively, *n* is the refractive index of the solvents, *D* is the integrated intensity, and Φ is the luminescence quantum yield. The quantity *B* was calculated by *B* = 1 – 10^–*AL*^, where *A* is the absorbance at the excitation wavelength and *L* is the optical path length.

### Hg^2+^ detection in aqueous buffered solution

Complex **1** (0.5 µM) and different concentrations of Hg^2+^ ions were added into Tris-HCl buffer (25 mM Tris, pH 7.0). Emission spectra were recorded in the 390−620 nm range using an excitation wavelength of 310 nm.

## Supporting Information

Figure S1 Emission and excitation spectrum of complex **1** (20 µM) in acetonitrile solution at 298K.
(TIF)

Figure S2 UV/Vis spectrum of complex **1** (20 µM) in acetonitrile solution at 298 K.
(TIF)

Figure S3 Relative intensity change at 490 nm of various concentrations of complex **1** in Tris-HCl buffer (25 mM Tris, pH 7.0) with the same concentration of Hg^2+^ ions (30 µM).
(TIF)

Figure S4 Emission spectrum of complex **1** (0.5 µM) upon addition of Hg^2+^ (30 µM) and upon subsequent addition of cysteine (0–80 µM) in buffered solution (25 mM Tris, pH 7.0).
(TIF)

Figure S5 ^1^H NMR spectrum of 1 (5 µM, upper panel) in the absence or in the presence of Hg^2+^ ions (500 µM, lower panel).
(TIF)

Figure S6 Emission spectrum of complex **1** (0.5 µM) upon addition with mix  =  Pb^2+^, Fe^3+^, Co^2+^, La^3+^, Ti^3+^ (each 150 µM) and upon subsequent addition of Hg^2+^ (30 µM) in aqueous buffered solution (25 mM Tris, pH 7.0).
(TIF)

Table S1 Photophysical properties of the iridium complex **1.**
(DOCX)
