# Supplementary figures and images for: A Highly Selective and Non-Reaction Based Chemosensor for the Detection of Hg2+ Ions Using a Luminescent Iridium(III) Complex
Source: PLoS One. 2013 Mar 22;8(3):e60114. doi: 10.1371/journal.pone.0060114 (PMC3606269; doi:10.1371/journal.pone.0060114)

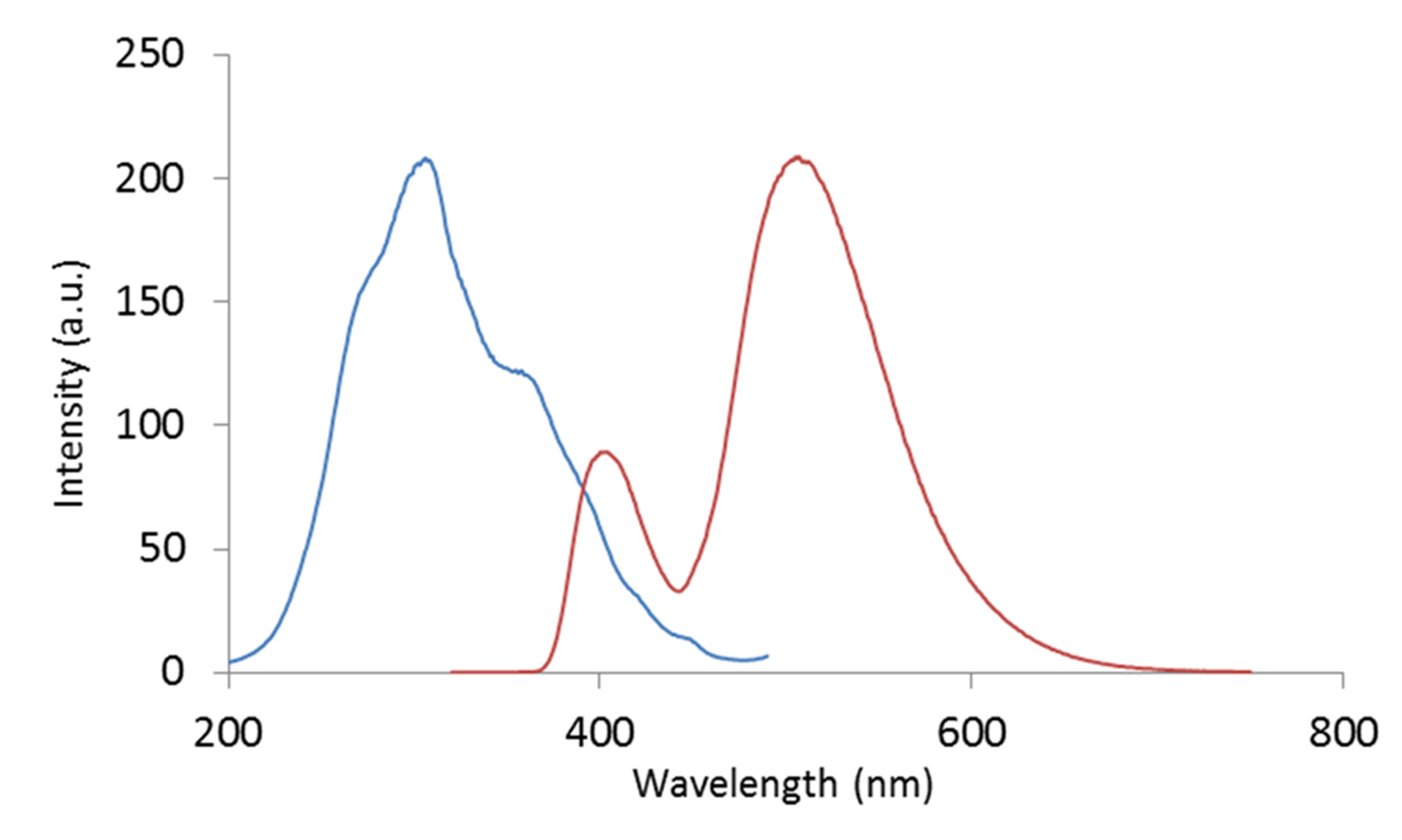

Supplement: Figure S1 — Emission and excitation spectrum of complex 1 (20 µM) in acetonitrile solution at 298K. (TIF) [file pone.0060114.s001.tif]

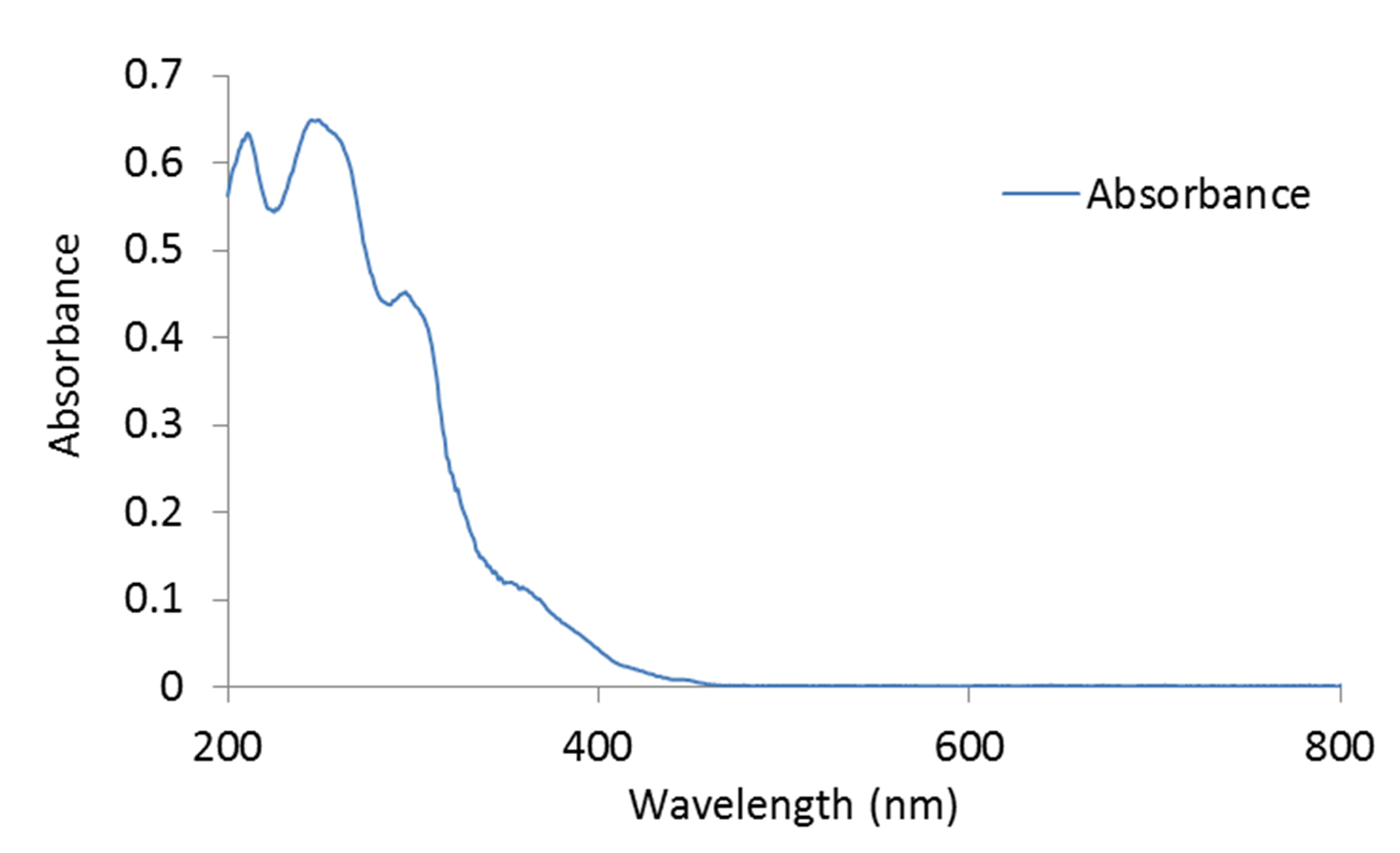

Supplement: Figure S2 — UV/Vis spectrum of complex 1 (20 µM) in acetonitrile solution at 298 K. (TIF) [file pone.0060114.s002.tif]

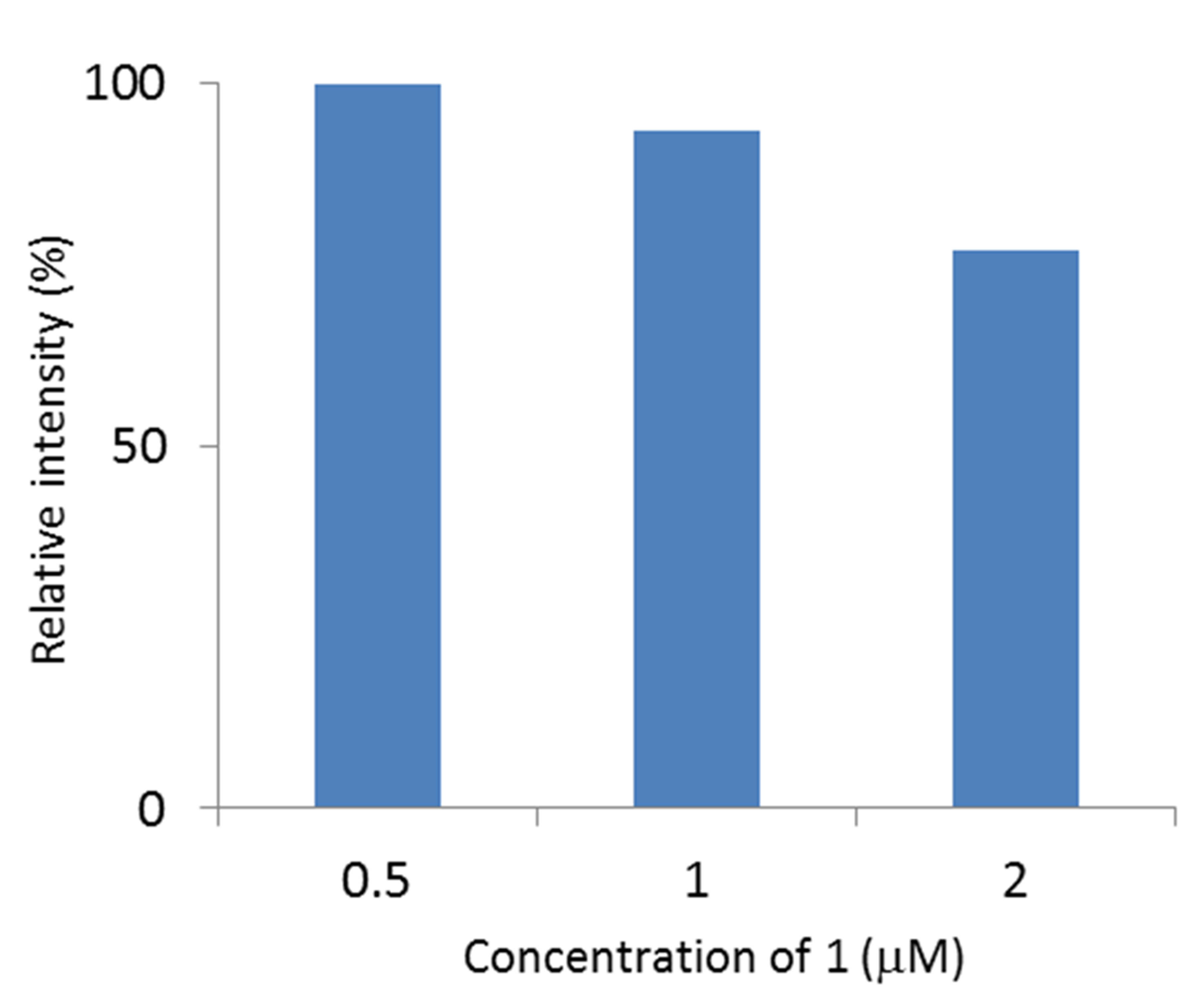

Supplement: Figure S3 — Relative intensity change at 490 nm of various concentrations of complex 1 in Tris-HCl buffer (25 mM Tris, pH 7.0) with the same concentration of Hg2+ ions (30 µM). (TIF) [file pone.0060114.s003.tif]

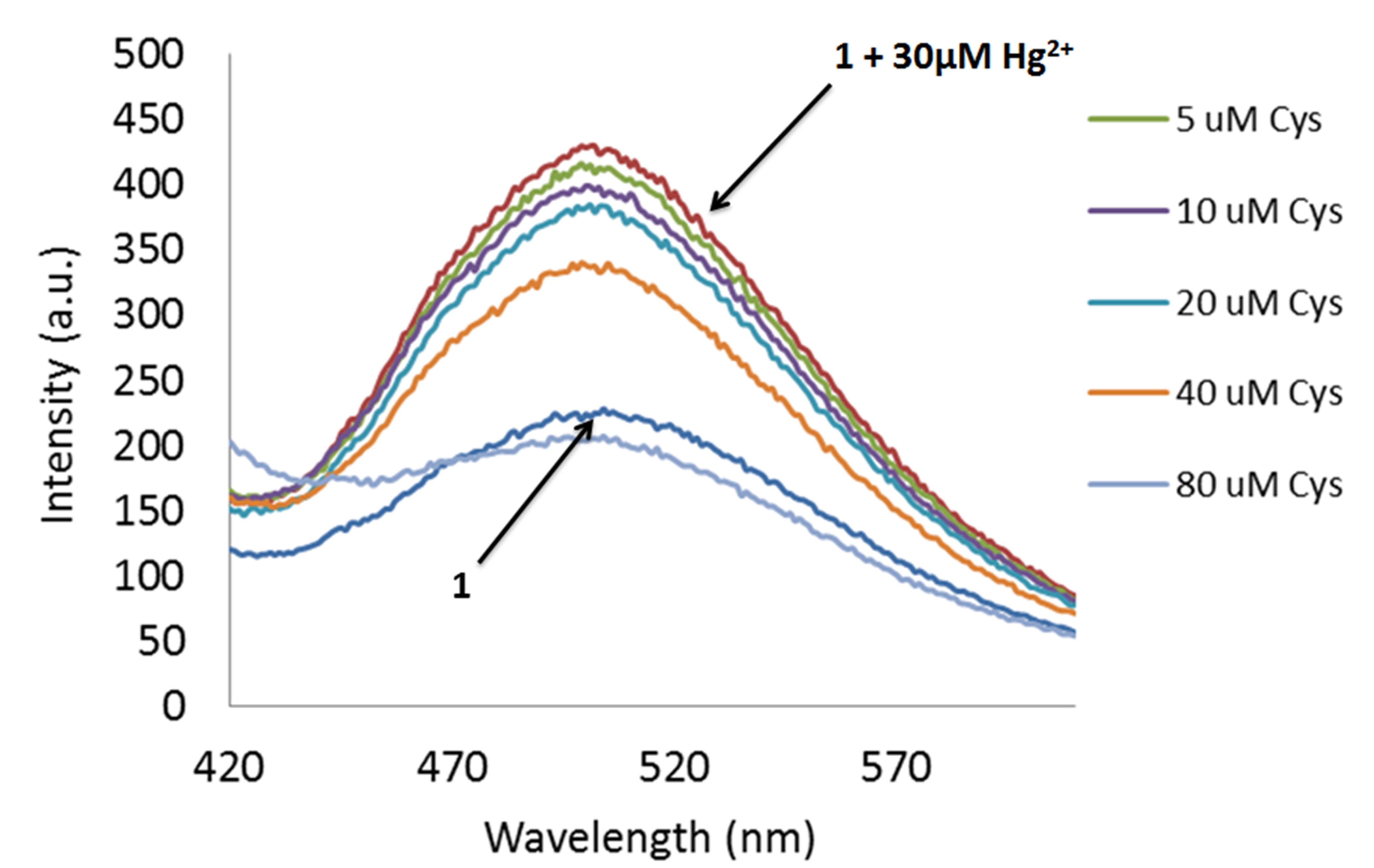

Supplement: Figure S4 — Emission spectrum of complex 1 (0.5 µM) upon addition of Hg2+ (30 µM) and upon subsequent addition of cysteine (0–80 µM) in buffered solution (25 mM Tris, pH 7.0). (TIF) [file pone.0060114.s004.tif]

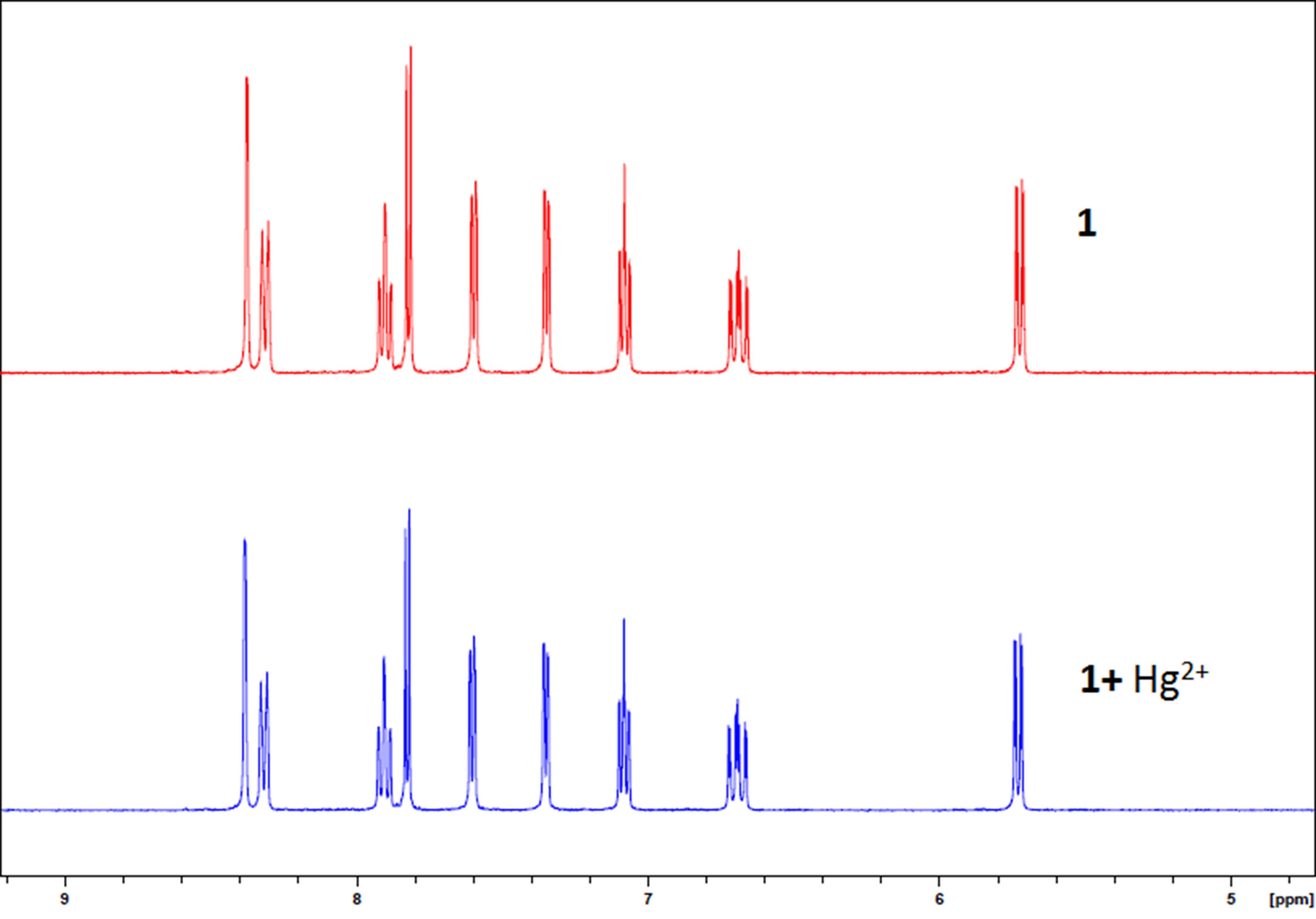

Supplement: Figure S5 — 1H NMR spectrum of 1 (5 µM, upper panel) in the absence or in the presence of Hg2+ ions (500 µM, lower panel). (TIF) [file pone.0060114.s005.tif]

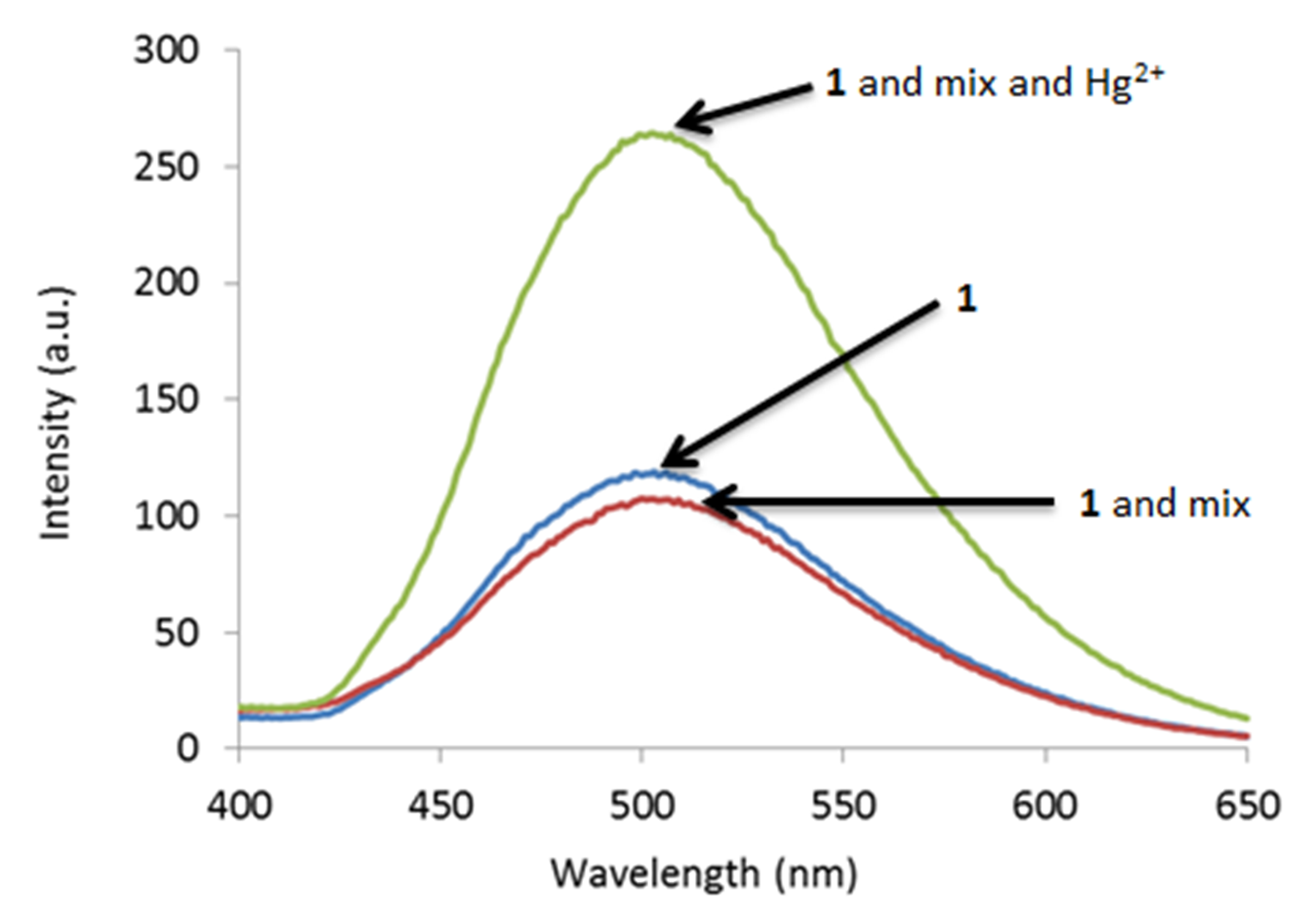

Supplement: Figure S6 — Emission spectrum of complex 1 (0.5 µM) upon addition with mix = Pb2+, Fe3+, Co2+, La3+, Ti3+ (each 150 µM) and upon subsequent addition of Hg2+ (30 µM) in aqueous buffered solution (25 mM Tris, pH 7.0). (TIF) [file pone.0060114.s006.tif]
