# Supplementary material for: A Highly Selective and Non-Reaction Based Chemosensor for the Detection of Hg2+ Ions Using a Luminescent Iridium(III) Complex
Source: PLoS One. 2013 Mar 22;8(3):e60114. doi: 10.1371/journal.pone.0060114 (PMC3606269; doi:10.1371/journal.pone.0060114)
Supplement: Table S1 — Photophysical properties of the iridium complex 1. (DOCX) [file pone.0060114.s007.docx]

**Table S1**. Photophysical properties of the iridium complex **1** ^a^

| Complex | UV-vis  λ/ nm ( dm^3^ mol^–1^ cm^–1^) | λ_max_  (nm) | φ | τ(µs) |
| --- | --- | --- | --- | --- |
| [Ir(dfppy)_2_(dnbpy)]PF_6_  (**1**) | 211 (3.06 x 10^4^)  255 (3.18 x 10^4^)  300 (2.19 x 10^4^)  386 (sh)  450 (sh) | 490 | 0.10 | 4.53 |

^a^ 50 μM solutions of complex **1** in degassed CH_3_CN at room temperature. Quantum yields were determined using [Ru(bpy)_3_]PF_6_ as reference.
